# *BRAF *and *RAS *oncogenes regulate Rho GTPase pathways to mediate migration and invasion properties in human colon cancer cells: a comparative study

**DOI:** 10.1186/1476-4598-10-118

**Published:** 2011-09-23

**Authors:** Eleni Makrodouli, Eftychia Oikonomou, Michal Koc, Ladislav Andera, Takehiko Sasazuki, Senji Shirasawa, Alexander Pintzas

**Affiliations:** 1 Laboratory of Signal Mediated Gene Expression, Institute of Biological Research and Biotechnology, National Hellenic Research Foundation, Vas. Constantinou Ave. 48, 11635, Athens, Greece; 2 Laboratory of Cell Signaling and Apoptosis, Institute of Molecular Genetics, v.v.i., Czech Academy of Sciences, 1083 Videnska, CZ-14220 Prague 4, Czech Republic; 3 Department of Pathology, International Medical Center of Japan, Tokyo, Japan; 4 Department of Cell Biology, Faculty of Medicine, Fukuoka University, Fukuoka, Japan

## Abstract

**Background:**

Colorectal cancer is a common disease that involves genetic alterations, such as inactivation of tumour suppressor genes and activation of oncogenes. Among them are RAS and BRAF mutations, which rarely coexist in the same tumour. Individual members of the Rho (Ras homology) GTPases contribute with distinct roles in tumour cell morphology, invasion and metastasis. The aim of this study is to dissect cell migration and invasion pathways that are utilised by BRAF^V600*E *^as compared to KRAS^G12V ^and HRAS^G12V ^oncoproteins. In particular, the role of RhoA (Ras homolog gene family, member A), Rac1 (Ras-related C3 botulinum toxin substrate 1) and Cdc42 (cell division cycle 42) in cancer progression induced by each of the three oncogenes is described.

**Methods:**

Colon adenocarcinoma cells with endogenous as well as ectopically expressed or silenced oncogenic mutations of BRAF^V600E^, KRAS^G12V ^and HRAS^G12V ^were employed. Signalling pathways and Rho GTPases were inhibited with specific kinase inhibitors and siRNAs. Cell motility and invasion properties were correlated with cytoskeletal properties and Rho GTPase activities.

**Results:**

Evidence presented here indicate that BRAF^V600E ^significantly induces cell migration and invasion properties *in vitro *in colon cancer cells, at least in part through activation of RhoA GTPase. The relationship established between BRAF^V600E ^and RhoA activation is mediated by the MEK-ERK pathway. In parallel, KRAS^G12V ^enhances the ability of colon adenocarcinoma cells Caco-2 to migrate and invade through filopodia formation and PI3K-dependent Cdc42 activation. Ultimately increased cell migration and invasion, mediated by Rac1, along with the mesenchymal morphology obtained through the Epithelial-Mesenchymal Transition (EMT) were the main characteristics rendered by HRAS^G12V ^in Caco-2 cells. Moreover, BRAF and KRAS oncogenes are shown to cooperate with the TGFβ-1 pathway to provide cells with additional transforming properties.

**Conclusion:**

This study discriminates oncogene-specific cell migration and invasion pathways mediated by Rho GTPases in colon cancer cells and reveals potential new oncogene-specific characteristics for targeted therapeutics.

## Background

Colorectal cancer represents a complex disease that involves multiple steps of genetic alterations, like inactivation of tumour suppressor genes and activation of oncogenes, often associated with progression from premalignant lesion (adenoma) to invasive adenocarcinoma [1]. *KRAS *mutations have been found in about 35% of colon carcinomas that mainly occur at codons 12, 13 and 61, resulting in a constitutively active form of KRAS GTPase. Consequently, multiple RAS effector pathways that regulate fundamental biological processes such as proliferation, apoptosis, and cell motility, become activated and/or deregulated. More specifically, mutant *KRAS *disrupts actin cytoskeleton and maintains motility in colon cancer cells [2]. Likewise, BRAF, a major downstream effector of KRAS, is also considered an oncogene whose activating mutations appear in 70% of human malignant melanomas and in about 12-18% of human colon cancers. The most frequent BRAF mutation is at codon 600 that results in elevated kinase activity [3,4]. Mutant *BRAF *may also interfere with organization of cytoskeleton and affect cell migration and invasion ability [5].

Key steps in invasion and metastasis are tightly regulated or influenced by the Rho family GTPases, which may include alterations in cell adhesion, cell-matrix, cell-cell interactions and actin organization, ultimately leading to the acquisition of an invasive phenotype. Many studies have investigated the role of Rho GTPases in tumour progression showing their contribution in cancer initiation and progression, through the acquisition of uncontrolled proliferation, survival and escape from apoptosis as well as tissue invasion and the establishment of metastasis [6]. Unlike *KRAS *and *BRAF*, mutations in *RHO *genes are extremely rare in tumours, but their expression and/or activity is frequently altered in a variety of human cancers. RhoA is frequently overexpressed in cancer [7], while depletion of Rac1 strongly inhibits lamellipodia formation, cell migration and invasion in carcinoma cells [8]. Another Rho family gene, Cdc42 is also important for cell motility and able to induce a mesenchymal-amoeboid transition in melanoma cells [9,10]. Regulation of Rho GTPases is extensively studied and it is well known that extracellular signal-regulated kinase (ERK) signaling is important for cell motility through Rho GTPases [2,11]. PI3K pathway is also involved in Rho family signal transduction and affects properties like cell migration [12,13]. Although a significant number of studies have analysed the role of Rho pathways in RAS-induced transformation, very little is known about the differential regulation of Rho GTPases by *RAS *and *BRAF *oncogene, as well as their subsequent contribution in oncogene-specific cell migration properties.

In order to invade into other tissues, epithelial cancer cells must disrupt the integrity of epithelium and basement membrane to enter the underlying stroma. This normally requires acquisition of a migratory phenotype, a process frequently referred as epithelial to mesenchymal transition (EMT). Invasive epithelial cancer cells often show reduced expression of E-cadherin, a cell-cell adhesion protein, and an increased expression of mesenchymal markers, such as vimentin and N-cadherin [14]. It has been shown that oncogenic HRAS is required for both induction and maintenance of EMT, mainly through its downstream effector ERK [15,16]. A representative model for studying EMT has been developed in our lab following stable transfection HRAS^G12V ^in colon adenocarcinoma Caco-2 cells (Caco-H cells). The transformation process rendered mesenchymal-like characteristics to the cells as determined by their morphology and global gene expression profile analysis [16,17].

Many regulators and effectors have been described for the Rho family GTPases that may be implicated in their functions, including Focal Adhesion Kinase (FAK), a protein known to contribute to EMT, and fascin that is mainly involved with actin cytoskeletal organization as well as cell migration, downstream of Rho GTPases [18]. Fascin is an actin-bundling protein normally upregulated in several epithelial neoplasms and may have prognostic value as an early biomarker for more aggressive colorectal adenocarcinomas, since it contributes to cancer cell migration *in vitro *and metastasis *in vivo *[19,20].

Since KRAS^G12V ^and BRAF^V600E ^mutations rarely coexist in human tumours, we aim to study their independent and comparative contribution in migration and invasion of colorectal cancer cells through Rho GTPases signalling. Towards this end Caco-2 cells, that represent an intermediate adenoma of human colorectal cancer, were stably transfected to ectopically express *KRAS^G12V ^*(Caco-K cells) and *BRAF^V600E ^*(Caco-BR cells) [21]. The doubling time and the cell cycle distribution by means of flow cytometry for each cell line have been examined. Results obtained indicated Caco-BR cells to have acquired a higher proliferation rate as compared to the parental cell line, Caco-2. For determining the "transformation potential", a number of cell properties were analyzed following stable transfection. *BRAF^V600E ^*induced cell properties, included altered morphology, colony formation ability in soft agar, tumorigenicity in SCID mice [21]. Here, we present evidence that BRAF^V600E ^enhances migration and invasion properties in colon carcinoma cells through RhoA activation, while KRAS^G12V ^induces these properties less efficiently as compared to BRAF^V600E^, albeit through Cdc42 activation and filopodia formation. In parallel, HRAS^G12V ^induces high migration and invasion ability through Rac1. These results indicate that although KRAS and BRAF are members of the same pathway, different Rho-dependent mechanisms are utilised by each oncogene to transform colon cancer cells. These findings could be exploited towards targeted therapies to Rho pathway components depending on the genetic background of the cancer patient.

## Materials and methods

### Cell culture

Caco-2, HT29 and DLD-1 human colon adenoma-carcinoma cell lines were obtained from American Type Culture Collection (ATCC) and DKO-4 cells were kindly provided by Drs T. Sasazuki and S. Shirasawa. Oncogenic models used in the present study were generated in Caco-2 cells by stable transfection in order to constitutively express HRAS^G12V ^(Caco-H), KRAS^G12V ^(Caco-K) or BRAF^V600E ^(Caco-BR) oncogenes and have been previously described [13,18]. In short, pcDNA3-KRAS^G12V^, pcDNA3-HRAS^G12V ^or pH8-BRAF^V600E ^plasmids were transfected into Caco-2 cells using the Ca_3_(PO_4_)_2 _precipitation technique and individual clones were selected with 0.5 mg/ml Geneticin (Sigma-Aldrigh, Poole, UK). All cell lines used in this study were grown in D-MEM medium supplemented with 10% FBS, antibiotics and amino acids. Caco-K6 and Caco-K15 clones were selected to overexpress KRAS^G12V^, Caco-H2 clones for overexpressing HRAS^G12V ^and Caco-BR13 and Caco-BR23 for overexpressing BRAF^V600E ^in Caco-2 cells. Since Caco-BR13 share similar properties with Caco-BR23 and likewise Caco-K6 are similar to Caco-K15, in some experiments data are presented only for Caco-BR13 and Caco-K15. In such cases the clones are named as Caco-BR and Caco-K respectively.

### Protein kinase inhibitors

Cells were treated with MEK inhibitor UO126 (Alexis Biochemicals, CA) to block MEK-ERK pathway. Wortmanin (Alexis Biochemical) was used to block PI3K pathway and for inhibition of RhoA/Rho kinase pathway the specific inhibitor Y-27632 (Sigma) was used. NSC23766 (Calbiochem, Gibbstown, US) was used to block Rac1 GTPase.

### Suppression of BRAF^V600E ^expression by RNA interference

HT29 cells were selected because of their *BRAF^V600E ^*mutation. The small inhibitory duplex shRNA oligo was cloned into the *Hind*III and *Bgl*II sites in pSUPER (Oligoengine, Seattle, WA). The sense strand of the shRNA pSUPER BRAF^V600E ^insert was BRAFmutshRNA: gatccccGCTACAGAGAAATCTCGAT-ttcaagagaATCGAGATTTCTCTGTAGCtttttggaaa {Hingorani, 2003 2/id}. HT29 cells were also transfected with the empty vector (pSUPER using the CaPO_4 _precipitation technique and selected with Geneticin (Invitrogen). The names of clones used in this study are: HTps (for empty vector) and HTshBR3 (for BRAF^V600E ^silencing).

### Western Blotting and GST pull-down assay

Whole cell lysates were prepared with lysis buffer containing 50 mM Tris-HCl (pH 7.4), 0,5% sodium deoxycholate, 0,1% sodium dodecyl sulfate (SDS), 500 mM NaCl, 10 mM MgCl_2_, 1% (v/v) Triton X-100, 1 mM sodium orthovanadate, 10 μg/ml aprotinin, 10 μg/ml leupeptin and 0.2 mM phenylmethylsulfonyl fluoride (PMSF). For Western blotting, protein concentrations were determined by the Bradford assay (Bio-Rad Laboratories, Hercules, CA) using bovine serum albumin as a standard. Extracts were resolved on SDS-PAGE (10% or 12% w/v acrylamide), transferred to nitrocellulose membrane (Whatman, Scheicher & Schuell, Dassel, Germany). Membranes were blocked with 5% milk in TBST for one hour and incubated with the specific antibodies overnight at 4°C, washed and incubated with the appropriate secondary antibody, for 1 hour at room temperature. Antibodies used were against: RhoA (sc-418), cdc42 (sc-87), Tubulin (sc-8035), Glyceraldehyde-3-phosphate dehydrogenase (GAPDH) (sc-47724), ERK2 (sc-1647), p-Cofilin (hSer 3) (sc-12912-R), Vimentin (sc-6260), E-cadherin (sc-7870), N-cadherin (sc-7939) and p-Myl (sc-12896-R) purchased from Santa Cruz Biotechnology (Santa Cruz, CA), Rac1 (05-389), FAK (05-537) purchased from Upstate (Lake Placid, NY, USA), pSer^445^B-Raf (2696) purchased from Cell Signalling (Danvers, MA, USA) and anti-fascin was a kind gift from Prof. Erik Langhoff. Antibody signal was obtained with the enhanced chemiluminescence plus Western blotting detection system (Amersham Biosciences, Uppsala, Sweden) after exposure to Kodak Super RX film. Values were measured using the Image-Quant software (Amersham Biosciences) and protein levels were normalized against tubulin.

For Rac1-GTP and cdc42-GTP GST pull-down assay, cells were cultured in 10 cm petri dish and cell lysates were prepared with lysis buffer used for western blotting. 500 μg of the identical protein extract were incubated with GST-PAK (p21-activated kinase) to glutathione agarose beads for 1 hour by rotating at 4°C and beads were washed 4 times in wash buffer (50 mM Tris-HCl (pH 7.4), 150 mM NaCl, 10 mM MgCl_2_, 1% (v/v) Triton X-100, 1 mM dithiothreitol (DTT), 10 μg/ml aprotinin, 10 μg/ml leupeptin and 0.2 mM PMSF). For RhoA-GTP GST pull-down assay it was used the Rho Assay Reagent [Rho-binding domain (RBD) of Rhotekin, agarose] from Upstate. All the experiments were repeated at least three times and representative images are shown.

### Immunofluorescence in cultured cells

Cells were grown on coverslips in 24-well plates and fixed using 4% paraformaldehyde (PFH) in PBS for 10 minutes at room temperature or cold methanol/acetone (8:1) for 10 minutes at -20°C. Cells that were fixed PFH were permeabilized with 0.1% Triton X-100 for 10 minutes shaking at room temperature. Cells were blocked with 4% fetal bovine serum (FBS) in phosphate-buffered saline (PBS) at room temperature for 1 hour and stained with the primary antibodies overnight at 4°C. Secondary antibodies Alexa Fluor 488 goat anti-mouse (A11001) or anti-rabbit (A11008) (Molecular Probes, Eugene, OR, USA) were applied to the cells for 1 hour at room temperature. For actin cytoskeleton staining cells were fixed with PFH, permeabilized and incubated with Alexa fluor phalloidin (A22283, Molecular Probes, Invitrogen). Nuclei were stained with Hoechst No. 33342 (Sigma, B2261) for 10 minutes at room temperature, coverslips were mounted on glass slides in Gelvatol/DABCO aqueous medium (Sigma-Aldrigh, Poole, UK) and visualized with a Leica TCS SPE confocal laser scanning microscope. LAS AF software was used for image acquisition (Leica Lasertechnik, Heidelberg, Germany).

### RNA Extraction/Reverse Transcription and Real Time-PCR

Total RNA isolation from cultured cells was performed using the Trizol reagent (Invitrogen, Karlsruhe, Germany). Reverse transcription was carried out from 3.0 μg of purified RNA using the SuperScript Reverse Transcriptase (Invitrogen, Karlsruhe, Germany) following the manufacturer's instructions.

Real-time quantification at the mRNA level was carried out in 96-well PCR plates using a Bio-Rad iCycler and the iQ5 Multicolor real-Time PCR detection system (Bio-Rad, Hercules CA, USA). Each reaction contained 1 × iQ SYBR Green Supermix (Bio-Rad, Hercules CA, USA) and 150 nmol/L of each primer. All genes were tested in triplicates. Results were analyzed on the iCycler software. Values were normalized to GAPDH. Primers used were the following: glyceraldehyde-3-phosphate dehydrogenase (**GAPDH**): GAAGGTGAAGGTCGGAGT (Fw) and CATGGGTGGAATCATATTGGAA (Rv), **E-cadherin: **GAACAGCACGTACACAGCCCT (Fw) and GCAGAAGTTCCCTGTTCCAG (Rv). **Ν-cadherin**: (Fw) TATATGCCCAAGACAAAGAGAC and TTCTGCTGACTCCTTCACTG (Rv), **Vimentin: **CTCGGTGGACTTCTCGCTGGCC (Fw) and TCCTGCAGGTTCTTGGCAGCC (Rv).

### Transwell Assays for Cellular Migration, Invasion and wound healing

For migration study, cells were trypsinised, washed thrice in medium with 1% FBS, and counted with a Z2 Coulter Counter (Beckman Coulter). Cells (1 × 10^4^) were plated into the upper chamber of 8 μm-pore Transwell filter (Corning) mounted in a 24-well dish with the lower chamber containing medium with 10% FBS. Before use, filters were pre-coated for 10 hours at 4°C with fibronectin (10 μg/ml; Sigma- Aldrich) and washed thrice. Cells were allowed to migrate in 5% CO2 for 30-36 hours at 37°C, fixed with methanol for 10 minutes at room temperature and stained with 0.1% crystal violet. The underside of the filters was examined with a 40 × objective of a Nikon Eclipse T-200 (Tokyo, Japan) inverted phase-contrast microscope and number of migrating cells was determined for each well. For cell invasion assay, the procedure was the same with the modification that the upper chamber was coated with Matrigel (Becton-Dickinson) and cells were let to invade through it. Each experiment was done 3 times in triplicates and measurements represent the average.

For wounding experiments, cells were plated in 24-well plates and allowed to grow to a confluency of 100%. Experimental wounds were made by dragging a Gilson plastic yellow pipette tip across the cell culture. Cultures were then rinsed with PBS and replaced with fresh culture media. Cell motility was then monitored at selected time points under the inverted light microscope.

### siRNA Transfection

Cells were transfected with human RhoA, Rac1, Cdc42, ROCK1 and ROCK2 ONTARGETplus SMART pool (Dharmacon) composed of four different duplexes (L-003860-00-0005, L-0035-60-00-0005, L-005057-00-0005, L-003536-00-0005 and L-004610-00-0005 respectively), or the siCONTROL RISC-free siRNA (D-001210-03; Dharmacon) using Invitrogen Lipofectamine™ according to the manufacturer's instructions. The day before transfection cells were plated into 6-well plates, so that they reached about 70% confluency the day of transfection. The amount of siRNA used was 160 pmol for Cdc42 and Rac1, 80 pmol for RhoA and 32 pmol for each ROCK1 and ROCK2 were applied in combination. Treatments with siRNA were replaced every 24 hours and western blot analysis verified the desired specific gene silencing 48 hours after transfection.

### 3D culture

For 3D culture experiments, cells were grown on coverslips in 24-well plates in medium with 5 mg/ml Matrigel. Briefly, 1 × 10^4 ^cells were mixed with the Matrigel-containing medium and a total volume of 300 μl was added in each well in order to form a gel of 1 mm thickness. Plates were placed in a cell incubator at 37°C for 1hour, so that gel was formed and 500 μl of complete medium was added on the top of it. Medium was changed every 2 days and cells left to grow for 12 days. Photographs of the 3D cultures were taken under light and confocal microscopes after the appropriate staining.

### Statistical analysis

Data are represented throughout the text with ± Standard deviation error bars. Statistical significance was tested with the unpaired Student *t*-test.

## Results

### BRAF^V600E ^induces distinct morphological changes in colon adenocarcinoma cells as compared to KRAS^G12V ^and loss of their epithelial architecture in 3D culture

**Figure 1:**
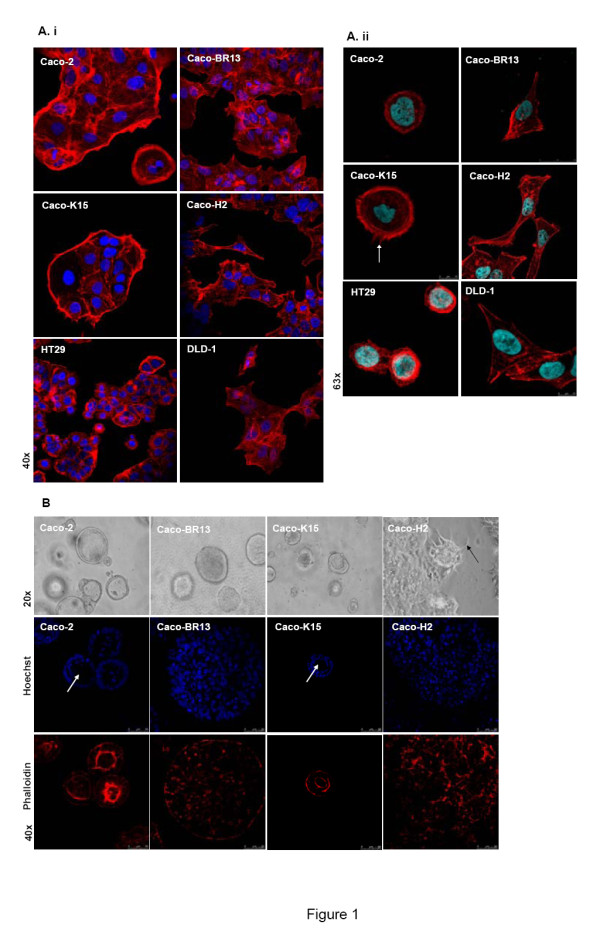
**BRAF^V600E ^induces loss of epithelial characteristics of colon adenocarcinoma cells**. (**A**) Confocal images after phalloidin staining show the altered morphology of Caco-BR13 cells and filopodia formation in Caco-K cells (arrow). Representative confocal images are shown in high (i) and low (ii) cell density. (**B**) Light microscope and confocal images of phalloidin staining in 3D cultures illustrates the architecture of Caco-2 and derived oncogenic models after 12 days of growth in Matrigel. Caco-2 and Caco-K15 cells formed spheroids with the typical lumen (arrows), a structure completely absent in Caco-BR13 and Caco-H2 cells.

Previously established Caco-BR cells have adopted a substantially different morphology when compared to the parental Caco-2 cells [21]. The elongated morphology acquired by Caco-BR cells was characterized by long membrane protrusions (Figure 1A, Additional Figure 1). We present evidence that the morphology of Caco-BR13 cells show properties of both Caco-2 epithelial nature and of the mesenchymal phenotype of Caco-H2 cells. On the other hand, Caco-K15 cells, which overexpress KRAS^G12V^, have retained the overall parental morphology of Caco-2 cells. For comparison, established adenocarcinoma cell lines HT29 and DLD-1, bearing mutant *BRAF^V600E ^*and *KRAS^G13D ^*respectively, have also been analyzed in the present study. It is of interest that the phenotype of Caco-BR cells resembles that of DLD-1 cells (KRAS^G13D^), especially since both of these cell types share high levels of p-BRAF (later analyzed). Our previous study shows important similarities between Caco-BR and DLD-1 cells regarding their tumourigenic properties and signaling pathways, suggesting that their transformation process occurs mainly through the constitutive activation of the MAPK (Mitogen-Activated Protein Kinase) pathway [21]. Staining with phalloidin resolved the morphological differences within the cell line panel indicating major actin cytoskeleton changes (Figure 1A). More specifically, in Caco-BR13 cells the formation of stress fibers was enhanced, whereas formation of filopodia-membrane protrusions enriched with actin- is evident in Caco-K15 cells (Figure 1A-ii, arrow).

In order to study in depth the morphology and architecture of the different cell lines under conditions that resemble the real tissue microenvironment, the three-dimensional (3D) culture system was adopted. As also previously shown [22], Caco-2 cells were organized into cyst-like structures that resemble normal colon cell architecture following their growth in Matrigel for about 12 days (Figure 1B). In contrast, Caco-H cells (EMT model) formed invasive masses with elongated protrusions, an architecture not shared by Caco-BR13 and Caco-K15 cells (Figure 1B, upper panel, black arrow). During 3D culture conditions, normal epithelial cells are organized into spheroids presenting a characteristic centrally-localized hollow lumen and distinct polarization of cells surrounding this lumen. Epithelial cancer cells do not form such structures; instead they develop non-polarized clusters with limited differentiation [23,24]. Following staining with Hoechst and phalloidin the ability of Caco-2 cells to form spheroids with lumen was observed, a property also retained by Caco-K15 cells but completely absent in Caco-BR13 and Caco-H2 cells (Figure 1B, lower panel). Significantly enlarged and more compact spheroids without lumen were formed by Caco-BR13 cells as compared to Caco-2 cells. In the case of Caco-H2 cells, no typical spheroids were formed, instead large masses with non-canonical shape were observed, typical of cancer cells. Therefore, under 2D as well as 3D culture conditions BRAF^V600E ^overexpression managed to alter the morphology of colon adenocarcinoma cells, rendering them a more mesenchymal-like phenotype, while KRAS^G12V ^conserved the epithelial architecture of Caco-2 cells in general.

### BRAF^V600E ^downregulates E-cadherin at the mRNA level and impairs its distribution in human colon adenocarcinoma cells

**Figure 2:**
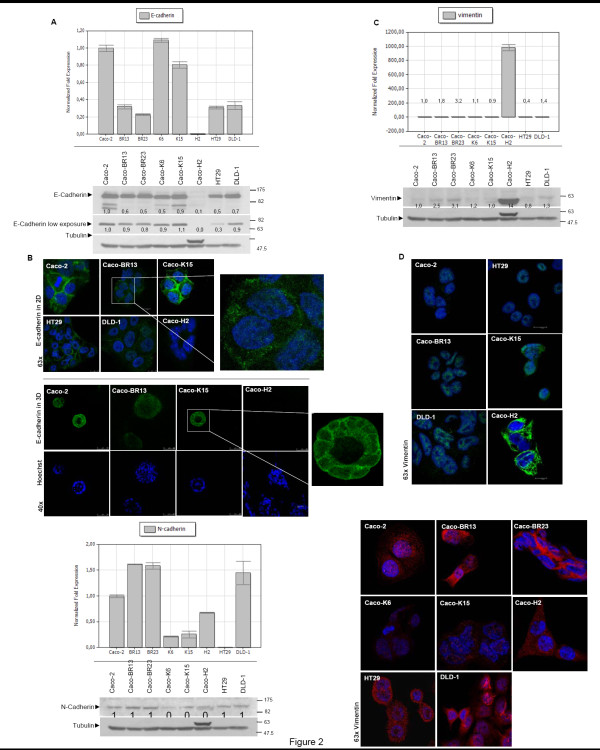
**BRAF^V600E ^impairs distribution of E-cadherin**. (**A**) Real-Time PCR (upper panel) and western blot analysis of E-cadherin (lower panel). (**B**) Confocal images in 2D (upper panel) and 3D (lower panel) conditions indicate disorganized of E-cadherin localization and its significant reduction in Caco-BR13 cells. (**C**) Real-Time PCR (upper panel) and western blot analysis of Vimentin (lower panel). (**D**) Confocal images of Vimentin show no alteration of this protein in Caco-BR13 and Caco-2 cells. (**E**) Real-Time PCR (upper panel) and western blot analysis (lower panel) for N-cadherin indicates augmentation of this mesenchymal marker in Caco-BR cells. (**F**) Confocal images of N-cadherin. Indicated antibodies in the western blot analysis were hybridized on the same membrane and slower migrating band appearing above tubulin represents Vimentin. Real-Time PCR expression experiments were performed twice in triplicates and representative confocal images are shown.

It has been previously shown that HRAS^G12V ^converts Caco-2 epithelial into mesenchymal cells by inducing loss of E-cadherin and overexpression of vimentin [25]. In order to examine whether BRAF^V600E ^had a similar effect on Caco-2 cells, the expression and localization of E-cadherin was analyzed (Figure 2A, B). Transformation of Caco-2 cells with BRAF^V600E ^led to a significant decrease in the mRNA levels of E-cadherin but had no significant effect on the actual protein expression (Figure 2A). Notably, in Caco-BR cells reduced intensity for E-cadherin was observed mostly in lower molecular weight protein bands representing the mature protein at 120 kDa (Figure 2A-lower panel, high exposure), whereas the decrease in the actual precursors at 135 kDa (Figure 2A-lower panel, low exposure), is considerably less. It appears that mutant BRAF^V600E ^but not upstream KRAS^G12V ^activation is able to suppress the mature E-cadherin, while the precursor remained mostly unaffected. Nevertheless, immunostaining with E-cadherin revealed a significant impairment of its distribution at the cell-cell boundaries since staining appeared discontinuous at the adherent junctions (Figure 2B, upper panel magnification). Expression of E-cadherin in the Caco-BR grown in 3D spheroids was found significantly downregulated with diffused distribution (Figure 2B, lower panel). In contrast, the epithelial marker E-cadherin was normally localized at the cell-cell junctions of Caco-2 and Caco-K15 cells (Figure 2B, lower panel magnification). In order to determine whether Caco-BR cells have acquired more mesenchymal characteristics, RNA and protein levels of the mesenchymal marker Vimentin were examined (Figure 2C). An increase of about 3-fold was observed at the protein level, while confocal images did not show significant difference, as compared to Caco-2 (Figure 2D), since it is known that some cancer epithelial cells abnormally express N-cadherin which has been shown to promote motility and invasion [26,27], N-cadherin expression was examined (Figure 2E). In Caco-BR cells N-cadherin expression is increased about 2-fold both at mRNA and protein levels, as compared to Caco-2 cells. Confocal images confirmed this increase, as shown in Figure 2F. Taken together these data suggest that BRAF^V600E ^overexpression failed to induce an integrated EMT phenotype, which is the case with HRAS^G12V ^overexpression [25], but managed to transform Caco-2 cells through the loss of some important epithelial characteristics.

### Differential BRAF^V600E^, KRAS^G12V ^and HRAS^G12V ^effect on the migration and invasion ability of Caco-2 cells *in vitro*

**Figure 3:**
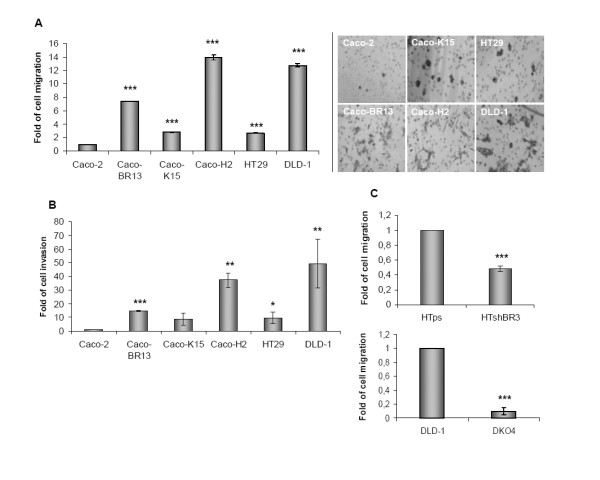
***KRAS^G12V^, BRAF^V600E ^*and *HRAS^G12V ^*oncogenic effects on the ability of colon adenocarcinoma cells to migrate and invade *in vitro***. (**A**) Cells were seeded on a polycarbonate membrane and allowed to migrate for 30 hours. Cells that migrated through the surface of the membrane were stained with crystal violet and counted by bright field microscopy. (**B**) For the invasion assay cell were seed on polycarbonate membrane coated with matrigel and incubate for 36 hours. (**C**) HT29 cells transfected with shRNA-BRAF^V600E ^(HTshB3) or empty vector (HTps) and DLD-1 cells before and after knockout of KRAS^G13D ^(DKO4). Migration assay was performed as mentioned in (A). *: *P *≤ 0,05; **: *P *≤ 0,01; ***: *P *≤ 0,001 as determined with Student *t*-test.

To further explore oncogenic effects on the cell cytoskeleton with regard to oncogenic transformation, the invasive and migratory properties of the previously established oncogenic cell models and in colon cancer cell lines HT29 and DLD-1 were analyzed. Transformation induced by each of the three oncogenes *KRAS^G12V ^*(Caco-K cells), *BRAF^V600E ^*(Caco-BR cells) and *HRAS^G12V ^*(Caco-H cells) managed to increase the ability of Caco-2 cells to migrate and invade *in vitro*, independently of their proliferating ability, which has been previously analyzed in [21]. More specifically, *BRAF^V600E ^*and *HRAS^G12V ^*provided Caco-2 cells with highly migrating and invasive properties, some similar to those in DLD-1 cells (Figure 3A, B), which is compatible with their more elongated morphology described earlier (Figure 1). Moreover, Caco-K cells, that retained typical epithelial morphology of Caco-2 parental cells also presented enhanced migrating and invasive properties, but to a lesser extent. Taken together, morphological properties induced by either *BRAF^V600E ^*or *KRAS^G12V ^*oncogene affected the ability of Caco-2 cells to migrate and invade *in vitro*, but were not sufficient to fully reverse their epithelial phenotype. The role of *BRAF *and *KRAS *oncogenes in altering cytoskeletal properties was further emphasized following depletion of BRAF^V600E ^by shRNA in HT29 cells, where migration ability of HT-ShBR3 cells, with downregulated expression of mt*BRAF *gene, was significantly impaired as compared to the empty vector control HT-ps cells. Likewise, knock out of KRAS^G13D ^in DLD-1 cells (DKO-4) [28] significantly reverted the migration ability of DLD-1 cells (Figure 3C).

### BRAF^V600E ^enhances the ability of Caco-2 cells to migrate and invade *in vitro *through RhoA activation

**Figure 4:**
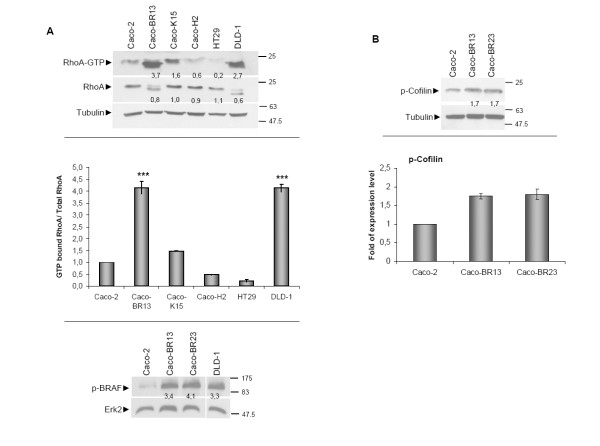
***BRAF^V600E ^*enhances the migrating and invasive capacity of colon adenocarcinoma cells through RhoA activation**. (**A**) GST-pull down assay for RhoA activation and quantification (upper and middle panel) and western blot analysis of pSer^445^BRAF levels in a panel of cell lines (lower panel). (**B**) Western blot analysis p-Cofilin that is enhanced in Caco-BR13 and Caco-BR23 cells as compared to the parental Caco-2 cells (upper panel) and quantification (lower panel).

Overexpression of BRAF^V600E ^in Caco-2 cells had a profound effect on the RAS effector protein RhoA (Figure 4). In Caco-BR cells activation of RhoA is increased (Figure 4A) as well as phosphorylation of its downstream target Cofilin, a protein that is related to stress fibre formation (Figure 4B). These findings are closely related to the observation regarding increased stress fibre formation indicated by phalloidin staining in Caco-BR13 cells (Figure 1). Notably, an extra band of lower molecular weight is detected for RhoA in Caco-BR and DLD-1 cells, which potentially represents the main active GTPase form (Figure 4A). A variant of lower molecular weight for RhoA protein has previously been reported both in colon and breast tissues [7]. However, RT-PCR analysis and treatment with the proteasome inhibitor MG-132, both in Caco-BR and DLD-1 cells, suggested no association of this faster migrating RhoA band with alternative splicing or proteasomal degradation (data not shown). These data suggested that the additional band potentially represents a post-translational modification of RhoA protein. To further explore the role of BRAF^V600E ^in the activation of the RhoA pathway, transient transfection of the oncogene in Caco-2 cells was performed (Additional Figure 2). Subsequent analysis of the migration and invasion properties showed that moderate RhoA activation induced a partial cell migration and cell invasion response (Addition Figure 2A, B). Notably in the invasion assay cell phenotype became slightly altered and resembled that of the stable Caco-BR clones (Additional Figure 2C), suggesting that a stable expression of BRAF^V600E ^is required to achieve complete cell transformation and extensive RhoA activation.

**Figure 5:**
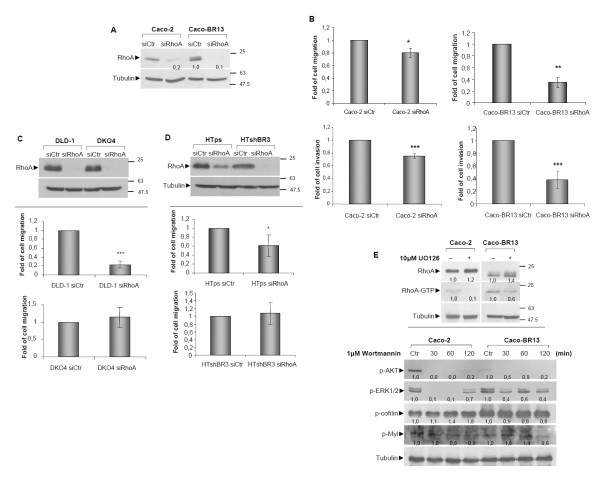
**The impact of depleting RhoA in cell migration and invasion and its regulation by signalling pathways**. (**A**) Caco-2 and Caco-BR cells treated for 48 hours with 80 pmol of a negative siRNA control sequence (siCtr) or with siRNA against RhoA and analysed by western blot with indicated antibodies. (**B**) Cell migration and cell invasion of Caco-2 and Caco-BR13 following siRNA of RhoA. (**C**) DLD-1 and DKO4 cells treated with 80 pmol of siRNA against RhoA for 48 hours and analyzed by western blot for RhoA. (upper panel). Cell migration ability was assayed 48-hour after treatment with siRNA against RhoA (lower panel). (**D**) HTps, HTshBR3 cells treated with 80 pmol of siRNA against RhoA for 48 hours and analyzed by western blot for RhoA (upper panel). Cell migration ability was assayed 48-hour after treatment with siRNA against RhoA (lower panel). (**E**) GST pull-down RhoA activation assay following treatment for 30 minutes with 10 μM of UO126 was performed in Caco-2 and Caco-BR13 cells (upper panel). Levels of p-cofilin and p-Myl were analysed by western blot following treatment with 1 μM wortmanin for 30-120 minutes (lower panel). *: *P *≤ 0,05; **: *P *≤ 0,01; ***: *P *≤ 0,001 as determined with Student *t*-test.

Regarding the importance of RhoA activation in the induced cell migration and invasion observed in Caco-BR cells, siRNA against RhoA was performed leading to significant protein depletion in both Caco-2 and Caco-BR13 cells (Figure 5A). Depletion of RhoA substantially impaired both acquired properties with more profound effect in Caco-BR13 cells, further illustrating its central role in the BRAF^V600E ^oncogene-induced transformation of colon adenocarcinoma cells (Figure 5B). Moreover, following RhoA depletion in Caco-2 cells, the number and size of stress fibres were notably reduced as compared to Caco-BR cells, where no such alteration was observed (data not shown).

In order to study further the impact of RhoA GTPase on cell migration, silencing of RhoA was performed in DLD-1 and HT29 cells. Considering that these cell lines bear mutation in KRAS^G13D ^and BRAF^V600E ^respectively, RhoA depletion was also performed in selected clones where KRAS^G13D ^(DKO4) or BRAF^V600E ^(HTshBR3) was knocked out or down regulated via shRNA respectively. This approach can implement the connection between each oncogene and the small GTPase. After silencing of RhoA, cell migration was significantly reduced in DLD-1, while no reduction was observed in DKO4 cells, where mutant KRAS^G13D ^is knocked out, (Figure 5C). Depletion of RhoA in HTshBR3 (transfected with shRNA pSUPER BRAF^V600E^) cells with suppressed BRAF^V600E ^activity did not reverse the ability of HT29 cell to migrate, while in HTps (HT29 cells transfected with empty vector pSUPER) a moderate reduction in cell migration was observed (Figure 5D). Taken together, these results indicate that both *BRAF *and *KRAS *oncogenes utilize RhoA activation to promote cell migration.

In a different approach, inhibition of RhoA downstream signalling was achieved via treatment of cells with UO126, a MEK (mitogen-activated protein kinase/extracellular signal-regulated kinase kinase) inhibitor targeting the MAPK pathway, which is active in Caco-BR cells [21]. Treatment with UO126, at the most optimal treatment condition (Additional Figure 3), resulted in the decreased activation of RhoA illustrating that mutant BRAF^V600E ^utilises the MAPK pathway to activate RhoA (Figure 5E, upper panel). Alternative regulation of RhoA through the PI3K pathway was analysed in Caco-BR cells, and a mild effect on RhoA downstream components like p-Cofilin and p-Myl was observed (Figure 5E, lower panel).

### Analysis of RhoA-ROCK axis

**Figure 6:**
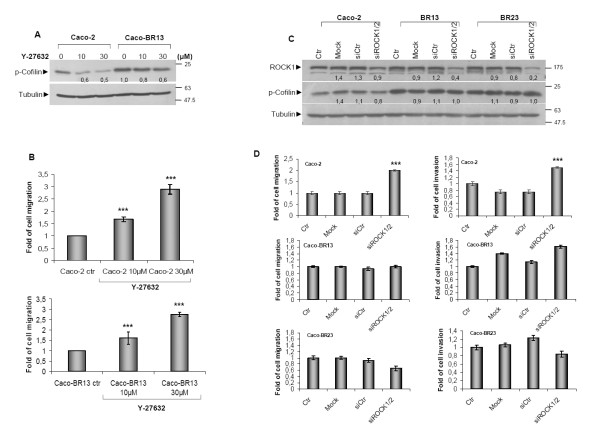
**Inhibition of RhoA-Rho kinase pathway in Caco-2 and Caco-BR cells**. (**A**) Caco-2 and Caco-BR13 cells were treated with the Rho kinase inhibitor Y-27632 for 4 hours at indicated concentrations to achieve downregulation of p-Cofilin. (**B**) The effect of Y-27632 on cell migration in Caco-2 and Caco-BR cells. ***: *P *≤ 0,001, as determined with Student *t*-test. (**C**) Cells were treated for 48 hours with 64 pmol of a negative siRNA control sequence (siCtr) or with 32 pmol siRNA against ROCK1 combined with 32 pmol against ROCK2. (**D**) Cell migration and invasion was determined following treatment with siRNA against ROCK1/2.

Since RhoA appears to be essential for the attained migration in Caco-BR13 cells, RhoA-Rho kinase signalling was inhibited using the selective ROCK (Rho-associated coiled coil forming protein serine/threonine kinase) inhibitor Y-27632 aiming to inhibit cell migration. Treatment of Caco-2 and Caco-BR13 cells with the ROCK inhibitor had a moderate effect on downstream target p-Cofilin, while cell motility was found significantly increased in both cell lines (Figure 6A-B). To exclude the possibility of this observation being the non-specific effect of the inhibitor targeting several other kinases, siRNA against both ROCK isoforms (ROCK1 and ROCK2) was applied to both Caco-BR clones and parental Caco-2 cells (Figure 6C). Besides, the use of siRNA to deplete a protein and especially a small GTPase can prove more promising since the specific protein sequence is targeted. In several reported studies, treatment with a selective inhibitor may produce more adverse effect through interaction with other (associated) components. Regardless efficient ROCK depletion, no inhibition in cell migration or invasion was observed in BRAF^V600E ^transformed cells (Figure 6D). Nevertheless increase motility was recorded in Caco-2 cells suggesting that Rac1 activation may be taking a lead role in the absence of the RhoA-Rho kinase signalling.

### KRAS^G12V ^induces Cdc42-dependent migration ability and filopodia formation in Caco-2 cells, partially dependent on PI3K pathway

**Figure 7:**
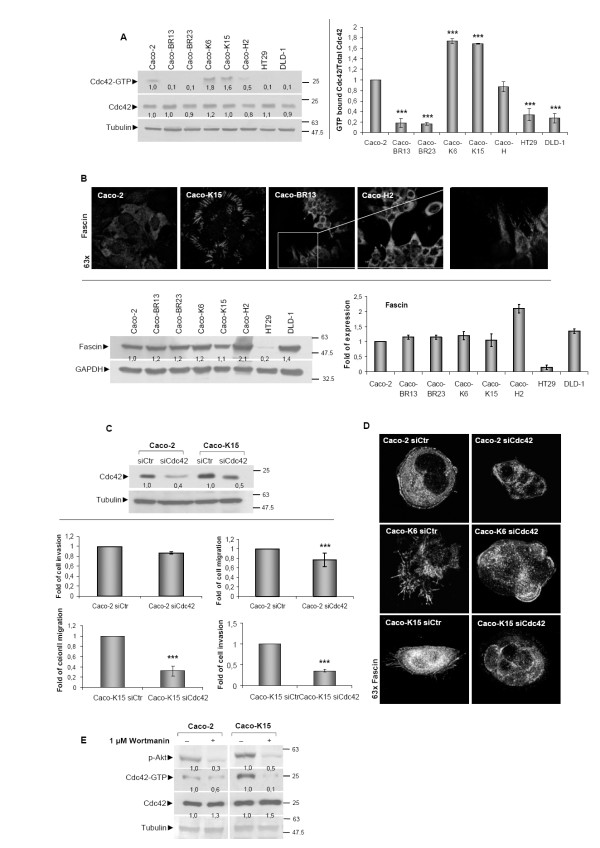
***KRAS^G12V ^*enhances migrating and invasive capacity of colon adenocarcinoma cells through Cdc42 activation**. (**A**) GST-pull down assay for Cdc42 activation in a panel of cell lines and quantification. (**B**) Fascin confocal staining illustrates filopodia formation (upper panel) and western blot analysis for expression of fascin and quantification (lower panel). (**C**) Caco-2 and Caco-K15 cells were treated for 48 hours with 160 pmol of a negative control siRNA sequence (siCtr) or with Cdc42-specific siRNA analyzed by western blot (upper panel). Assesment of cell migration and invasion in Caco-2 and Caco-K15 cells following siRNA against Cdc42 for 48 hours (lower panel). (**D**) Confocal images of Fascin in Caco-K cells after treatment with siRNA against Cdc42. (**E**) GST pull-down assay for Cdc42 activation in Caco-2 and Caco-K15 cells following treatment for 30 minutes with 1 μM PI3K inhibitor wortmanin. Results of migration and invasion assays were consistent between 3 independent experiments performed in triplicates. ***: *P *≤ 0,001 as determined with Student *t*-test.

Previous studies have indicated that RhoA, Rac1 and Cdc42 signalling is essential for oncogenic Ras transforming capacity [29,30]. In the present study, Caco-2 cells overexpressing mutant KRAS^G12V ^(Caco-K), selective activation for Cdc42 was detected (Figure 7A). The formation of filopodia in these cells, earlier described, was in agreement with the high Cdc42 activity and is illustrated here by staining with antibody against Fascin, a filopodia marker (Figure 7B, upper panel). A large number of relatively short filopodia distributed almost exclusively at the cell periphery was evident in Caco-K cells, while Caco-BR and Caco-H cells formed less but longer structures with a rather polarized shape potentially pointing towards the direction of cell migration (Figure 7B, upper panel). Nevertheless, no changes in Fascin protein expression were recorded in the different cell lines, (Figure 7B, lower panel). Increased migration ability in Caco-BR and Caco-H cells may be indicative for the length and the location of filopodia. It has been previously shown that in CHO-K1 cells (Chinese hamster ovary fibroblast- like cells) RhoA expression down-regulates Cdc42 and Rac1 activity in order to regulate membrane protrusions and cell polarity. In addition, Rac1 activity may down-regulate Cdc42 activity and promote the formation of stabilized rather than transient protrusions [31]. Indeed, low Cdc42 activity was recorded in Caco-BR and Caco-H cells where RhoA signaling is activated. To explore the role of Cdc42 in mutant KRAS^G12V ^induced cell transformation, Caco-2 and Caco-K15 cells were treated with siRNA against this small GTPase. Significant downregulation of Cdc42 at the protein level was observed in both cell lines (Figure 7C-upper panel), that caused a significant decrease of cell migration and invasion ability of Caco-K15 and of Caco-2 cells but to a lesser extent (Figure 7C-lower panel). Depletion of Cdc42 also affected the filopodia formation, when Caco-K cells were treated with siRNA against Cdc42 acquired rounded cell membrane lacking filapodia protrusion suggesting that filopodia formation in Caco-K cells is Cdc42-dependent (Figure 7D). These findings suggest that KRAS^G12V ^regulates motility and invasiveness of colon cancer cells through the Cdc42 GTPase.

Considering that the PI3K pathway is also a KRAS effector pathway, the possibility of a cross-talk between the PI3K signalling pathway and Cdc42 was explored [16,21]. Following treatment with wortmanin at the most optimal treatment condition, as retrieved from inhibition of the active PI3K pathway in Caco-H2 cells that show high p-AKT levels (Additional Figure 4), resulted in reduced Cdc42 activity. This illustrates how Cdc42 activation in response to the KRAS^G12V^-PI3K signalling pathway can be potentially essential for Cdc42-dependent cell migration and invasion properties (Figure 7E).

### HRAS^G12V ^induces high cell migration and invasion properties mediated by Rac1 associated with acquired EMT

**Figure 8:**
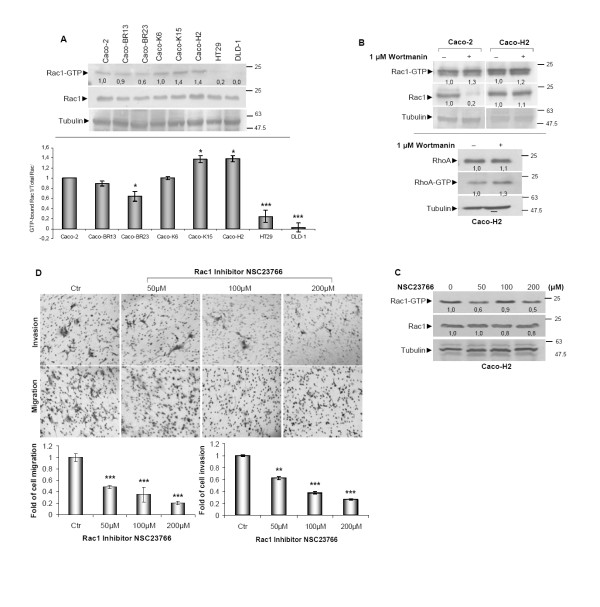
**Rac1 is important for cell migration and invasion properties of Caco-H cells**. (**A**) GST-pull down assay for Rac1 activation in a panel of cell lines (left panel) and quantification (right panel). (**B**) Caco-2 and Caco-H2 cells were treated with 1 μM PI3K inhibitor wortmanin for 30 minutes and GST pull-down assay for Rac1 activity (upper panel) and RhoA activity (lower panel) was performed. (**C**) Caco-H2 cells were treated with Rac1 specific inhibitor NSC23766 for 36 hours at indicated concentrations after which a GST-pull down assay for Rac1 activation was performed. (**D**) Cell migration and invasion ability of Caco-H2 cell in the presence of Rac1 inhibitor NSC23766 was estimated after 36 hours (upper panel) and quantification.(lower panel) *: *P *≤ 0,05 as determined with Student *t*-test.

Activation of Rac1, another RAS effector protein, was found slightly increased in Caco-H2 cells with EMT characteristics [15,25] (Figure 8A). Activation of Rac1 in Caco-H2 cells is in agreement with previous studies that correlate Rac1 with EMT and the inhibition of E-cadherin in mammary epithelial and pancreatic carcinoma cells respectively [32]. In contrast, a weak effect on Rac1 GTPase was recorded in Caco-BR cells (Figure 8A) and could be explained by the known antagonistic effect that exists between RhoA and Rac1 [33]. As described earlier, HRAS^G12V^-transfected Caco-2 cells (Caco-H2) have undergone EMT, followed by the dramatic reduction of E-cadherin expression [16]. Following PI3K pathway depletion using the specific inhibitor wortmanin at the most optimal treatment condition (Additional Figure 4), Rac1 activity was successfully inhibited only in Caco-2 cells, leaving Caco-H2 cells unaffected (Figure 8B, upper panel). Notably, under the same treatment conditions RhoA activity was found to be slightly increased, suggesting an involvement of the PI3K pathway in RhoA regulation (Figure 8B, lower panel). It is therefore concluded that in Caco-H2 cells, HRAS^G12V ^deregulates PI3K-dependent activation of Rac1 as well as mediates RhoA inhibition. To further explore the involvement of Rac1 activation in the transforming ability of HRAS^G12V ^in Caco-2 cells, pharmacological inhibition of Rac1 was established using the selective inhibitor NSC23766 (Figure 8C) [34]. Inhibition of Rac1 not only managed to suppress Rac1 activation but also to abolish cell migration and invasion properties in a dose dependent manner (Figure 8D), indicating the critical role of Rac1 in EMT cell properties of Caco-H cells.

### TGFβ-1 co-operates with *BRAF^V600E ^*and *KRAS^G12V ^*oncogenes to provide Caco-2 cells with enhanced transformation properties

**Figure 9:**
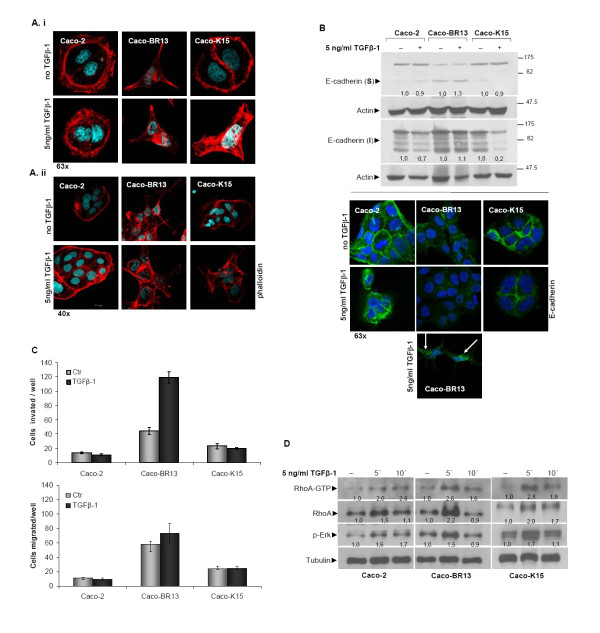
**TGFβ-1 enhances the transforming properties by *BRAF^V600E ^*and *KRAS^G12V ^*oncogenes**. (**A**) Confocal images of phalloidin staining after 14 days of TGFβ-1 treatment illustrate a more transforming phenotype acquired by Caco-K15 cells. Representative confocal images are shown in low (i) and high (ii) cell density. (**B**) Western blot analysis of E-cadherin in soluble and insoluble cell fractions (upper panel) and confocal images (lower panel) after 14 days of TGFβ-1 treatment. Fragmentation of E-cadherin in Caco-BR13 cells is indicated by arrows. (**C**) Assessment of cell migration and invasion of Caco-2, Caco-BR13 and Caco-K15 cells during TGFβ-1 treatment for 14 days. (**D**) Effect of TGFβ-1 in RhoA activation and ERK1/2 phosphorylation. Caco-2, Caco-BR13 and Caco-K15 cells were serum starved overnight and treated with 5 ng/ml TGFβ-1 for 5 and 15 minutes after which a GST pull-down RhoA activation assay was performed.

Since *BRAF^V600E ^*and *KRAS^G12V ^*oncogenes did not manage to fully transform Caco-2 cells nor induced an EMT phenotype, as *HRAS^G12V ^*did, it was further investigated whether co-operation of oncogene-growth factor can produce synergistic effect. The previously established oncogenic models of BRAF^V600E ^and KRAS^G12V ^along with the parental Caco-2 cells were treated with Transforming Growth Factor beta-1 (TGFβ-1) for 14 days. Staining with phalloidin revealed significant morphological changes in TGFβ-1 treated Caco-K15 cells that were not observed in Caco-2 cells following treatment with TGFβ-1, while no morphological changes were recorded in TGFβ-1 treated Caco-BR13 cells (Figure 9A). Protein analysis for E-cadherin, in fractionized soluble (intracellular) and insoluble (bound-membrane E-cadherin) extracts indicated a reduction of E-cadherin in the insoluble fraction in Caco-2 and Caco-K15 cells to a greater extend (Figure 9B, upper panel). Interestingly, even though levels of E-cadherin were not altered in Caco-BR13 cells, confocal images clearly presented disrupted cell-cell contacts and discontinuous staining which weakens cell junctions allowing cell migration (Figure 9B, lower panel-arrows). Altered localization of E-cadherin is an important mechanism contributing to cell metastasis [35].

TGFβ-1 was also investigated for its potential effect on cell migration and invasion. Treatment with TGFβ-1 increased the capacity of Caco-BR13 cells to invade *in vitro*, while no effect in the migrating ability of these cells was recorded (Figure 9C). This enhanced invasive capacity of Caco-BR13 cells is independent of their cell proliferation (Additional Figure 5). In contrast, cell migration and invasion of Caco-2 and Caco-K15 cells were not affected by TGFβ-1 treatment, although KRAS^G12V^-transfected cells acquired a more elongated morphology and slightly downregulated E-cadherin. Taken together, these results suggest that TGFβ-1 can synergise with KRAS^G12V ^and BRAF^V600E ^oncogenes to provide Caco-2 cells with a more transforming phenotype.

According to previous studies, the mutation in the C-terminal domain of Smad4, D351H, that is present in Caco-2 cells, results in complete Smad4 inactivation [36]. However, TGFβ-1 has been shown to act through alternative non-Smad pathways, such as Rho GTPases and MAPK [37-39]. Indeed, following TGFβ-1 treatment, enhanced activity for RhoA GTPase as well as pERK1/2 was recorded in Caco-2, Caco-K15 and Caco-BR13 cells. Based on these observations other than non-Smad signaling like RhoA GTPase and pERK1/2 pathways can be regulated by TGF-beta, to induce the morphological changes observed in the Caco-2 transformed and parental cells (Figure 9D).

## Discussion

### *BRAF^V600E^, KRAS^G12V ^*and *HRAS^G12V ^*oncogenes differentially modify morphology and epithelial characteristics of Caco-2 cells

As presented in this study, the three oncogenes induce different changes on cell morphology. Specifically, BRAF^V600E ^alters the typical epithelial morphology of Caco-2 cells, the distribution of E-cadherin and reduces its expression at the mRNA level. The elongated morphology that Caco-BR cells acquired lies between the epithelial of Caco-2 and the mesenchymal of HRAS^G12V^-transfected cells (Caco-H). However, the exact mechanism of this effect needs to be further investigated. There is evidence that Rho GTPases play role in regulation of E-cadherin. More specifically, active forms of Rac1 and Cdc42 have a positive effect on E-cadherin mediated cell-cell adhesions, while RhoA may also participate to a lesser extent [40]. On the other hand, KRAS^G12V ^does not alter the epithelial phenotype of the cells, but induces increased number of filopodia, actin rich finger-like protrusions, that are important for cell polarity and the direction of cell movement [41]. Regarding HRAS^G12V^, EMT cells (Caco-H) have an invasive morphology, well illustrated both in 2D and 3D cell culture conditions and loss of E-cadherin expression. It has been established that E-cadherin expression can be downregulated in epithelial tumours by a number of mechanisms related to the induction of EMT [42,43]. In this study, BRAF^V600E ^has provided Caco-2 cells with altered epithelial morphology and high migrating and invading capacity. High vimentin expression is not detected in Caco-BR cells, like in Caco-H with EMT characteristics. Instead, Caco-BR cells over-express another mesenchymal marker, N-cadherin. Taken together these data suggest that BRAF^V600E ^is able to relax cell-cell junctions by reducing E-cadherin expression and may drive colon epithelial cells to a more aggressive phenotype, while KRAS^G12V ^reserves their epithelial characteristics.

The doubling time and the cell cycle distribution by means of flow cytometry for each oncogene has been already described [21]. The increased proliferation rate of transformed cells may influence cell invasion, but this could not be the only reason for the enhanced invasive ability. Here we show that small GTPase pathways regulate cell migration and invasion, which do not clearly affect cell proliferation pathways in our system. More specifically, HRAS^G12V ^induces high proliferation rates as well as very aggressive cell migration and invasion properties associated with EMT phenotype; **BRAF^V600E ^**provides maternal cells with increased proliferation and with enhanced migration properties; **KRAS^G12V ^**despite the fact that does not substantially alter cell growth and proliferation, provides Caco-2 cells with increased filopodia formation and enhanced migration properties.

### BRAF^V600E^, KRAS^G12V ^and HRAS^G12V ^enhance migrating and invading capacity of Caco-2 cells, through different Rho pathway

The three oncogenes BRAF^V600E^, KRAS^G12V ^and HRAS^G12V ^managed to enhance migrating and invading capacity of Caco-2 cells, but to a different extent, with HRAS^G12V ^being more efficient. These cell properties seem to be dependent of cell morphology, since Caco-BR and Caco-H cells that are more elongated show high migration and invasion as compared to epithelial Caco-2 and Caco-K cells. Moreover, the three oncogenes also differ concerning the activation of individual Rho pathway responsible for cell migration and invasion. RhoA GTPase is highly activated in Caco-BR cells, resulting in their increased ability to migrate and invade *in vitro*. So far, little is known about the exact correlation between RAF kinases and Rho GTPases and their impact on human cancer progression. Two previous studies have shown cooperation between RAF and RhoA in epithelial cell transformation and in melanoma progression. More specifically, constitutive active Raf-1 and RhoA cooperate in order to transform rat intestinal epithelial cells, providing them with a spindle-like morphology, anchorage independent growth and capacity to form tumours in athymic nude mice [44]. In our system, *BRAF^V600E ^*induces constitutively high pRaf-1 levels and provides Caco-2 cells with new characteristics, including spindle-like morphology, anchorage independent growth and capacity to form tumours in athymic nude mice, albeit through high levels of pBRAF and pRaf-1 [21]. In a different study, human metastatic melanoma cells were treated with siRNA against BRAF^V600E ^and S-phase kinase-associated protein-2 (Skp-2), a positive regulator of RhoA, which resulted in both cell migration and invasion inhibition, suggesting that the BRAF-MAPK pathway and Skp-2-RhoA cascade can contribute to the invasive nature of melanoma [45]. A more recent study revealed that TGF-β-mediated activation of RhoA is required for efficient *BRAF^V600E ^*transformation of NIH3T3 cells [37]. Herein, we present for the first time that *BRAF^V600E^*-induced ability of human colon epithelial adenocarcinoma cells to migrate and invade *in vitro *is mediated by RhoA pathway.

In the case of KRAS^G12V ^transformed cells (Caco-K) as indicated from data presented here, the three small GTPases (RhoA, Rac1 and Cdc42) are differentially activated. Towards this end, KRAS^G12V^-transfected cells present increased number of filopodia, actin reach finger-like protrusions, that are regulated by Cdc42 GTPase [41] and are important for cell polarity, as well as for the direction of cell movement. In contrast to *BRAF *oncogene, *RAS *has been widely studied concerning its cooperation with Rho GTPases in cancer progression. Targeted silencing of Cdc42 exhibited the importance of this GTPase in motility and invasion of Caco-K cells, suggesting that KRAS^G12V ^induces migration and invasion properties in human colon cancer cells through activation of Cdc42.

Regarding HRAS^G12V^, it is evident that Rac1 plays an important role in EMT properties of Caco-H cells, since inhibition of this GTPase with specific inhibitor, resulted in decreased capacity of the cells to migrate and invade *in vitro*. It is worth mentioning that inhibition of Rac1 was also attempted using specific siRNA, but downregulation of Rac1 was not significant (data not shown). Although activation of Rac1 in Caco-H cells is moderate, as compared to Caco-2, activity of RhoA is reduced, potentially due to antagonistic action of RhoA and Rac1 in actin cytoskeleton organization [46].

### Regulation of Rho GTPases pathway differs in each case of oncogene transformation

#### a. BRAF^V600E ^and RhoA

In our system, cross talk between BRAF*^V600E ^*and RhoA is mainly mediated through MEK-ERK pathway, as indicated by cell treatment with a MEK inhibitor. Additional data which link *BRAF^V600E ^*to Rho signalling were recently derived from microarray analysis preformed with Caco-BR cells in our lab (Joyce T., et al., *Under Revision*). Global gene expression analysis revealed that RhoA-specific guanine nucleotide exchange factors (GEFs), like GEF11 (PDZ-RhoGEF) and GEF18 (p114-rhoGEF) [47,48] were upregulated in Caco-BR cells. This indicates that mutant BRAF can positively regulate RhoA activity by modulating the expression of its regulatory factors. Remarkably, as presented in a recent study, ERK can promote Rho dependent focal adhesion formation by suppressing p190A RhoGAP [49]. Nevertheless, in our system RhoA-ROCK axis does not appear to play crucial role in the enhanced cell migration and invasion properties, since inhibition of ROCK does not alter the capacity of Caco-BR cells to migrate and invade *in vitro*. In agreement with this data, previous studies have shown that treatment of human endometrial stromal cells (hESCs) and NIH 3T3 mouse fibroblasts with ROCK inhibitor Y-27632 resulted in enhanced cell motility [50,51]. A possible explanation may be the fact that RhoA has alternative effectors, such as Dia1 which was shown to be involved in RhoA-dependent cytoskeletal properties. In human colon cancer cells Dia1 can act downstream of RhoA to regulate the actin network [52]. Previous studies using HeLa or breast cancer cells showed that active RhoA is required for the induction of membrane ruffles in migrating cells also mediated by Dia1 and not ROCK [52,53]. Here, active RhoA may potentially act mainly through Dia1 and not ROCK to induce migration and invasion in Caco-BR cells and for that reason downregulation of ROCK may not affect these cell properties.

**Figure 10:**
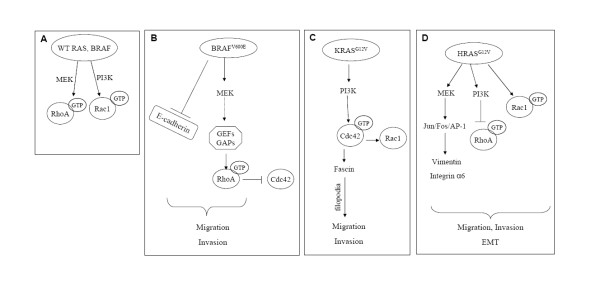
**Proposed models for BRAF^V600E^, KRAS^G12V ^and HRAS^G12V ^(EMT) - induced cytoskeletal changes in colon cells**. (**A**) Wild type RAS and BRAF regulate RhoA and Rac1; (**B**) BRAF^V600E ^regulates RhoA; (**C**) KRAS^G12V ^regulates Cdc42; (**D**) Implication of Rac1 signaling in HRAS^G12V^- induced EMT.

Notably, cross-talk analysis of small GTPases by means of selective siRNA revealed that RhoA may have an antagonistic function with Cdc42 in Caco-BR13 cells (Additional Figure 6A). This can be achieved though competition for common regulatory molecules, like Rho guanine nucleotide dissociation inhibitors (RhoGDIs) [54]. Based on these findings, a working model is proposed for BRAF^V600E^-induced invasive phenotype (Figure 10B): BAF^V600E ^induces MEK activation, which in turn activates RhoA most likely through specific GEFs and GAPs. In BRAF^V600E^-transformed cells, RhoA antagonises with Cdc42 through competition for common regulatory molecules. At the same time, E-cadherin is downregulated, resulting in the relaxation of cell-cell adhesion and increased migratory and invasive capacity. BRAF^V600E ^induced transforming properties are further enhanced through cooperation with TGFβ-1, suggesting that synergism between oncogene and growth factor is essential for induction of further migration properties in colon adenocarcinoma cells. Since Smad pathway is not functional in this cell system, due to an intrinsic mutation on Smad4 in Caco-2 cells, activation of RhoA in response to TGFβ-1 treatment, can potentially mediate the induced cell properties by TGFβ-1 related to EMT.

#### b. K-RAS, Cdc42 and PI3K pathway

In Caco-K cells, PI3K pathway is important for regulation of Cdc42 activity, as shown by treatment by specific PI3K inhibitors. According to another study, PI3K/Cdc42 and PI3K/Rac1 pathways are important in LPA-mediated migration of glioma cells [55]. Moreover, results from microarray analysis showed that in Caco-K cells Asef2, a guanine nucleotide exchange factor specific for Rac1 and Cdc42 is highly overexpressed (unpublished data). Remarkably, Cdc42 regulates Rac1 expression in KRAS^G12V ^stably expressing cells, since decreasing Cdc42 expression by specific siRNA results in downregulation of Rac1 in Caco-K15 cells (Additional Figure 6B). In a summarized model, downstream effectors of RAS constitutively active in response to KRAS^G12V^, such as PI3K or AKT, lead to activation of Cdc42 and Rac1 through specific GEFs. Active GTPase induces filopodia and lamellipodia formation that contribute in migration and invasion ability of the cells (Figure 10C). Although *KRAS^G12V ^*does not alter substantially the epithelial morphology of Caco-2 cells, its cooperation with TGFβ-1 induces a more aggressive phenotype indicating that this oncogene needs the contribution of a growth factor to accomplish cell transformation. Interestingly, mutant KRAS oncogene co-operates with TGFβ-1 to induce target genes like SNAIL, which regulates expression of E-cadherin in several systems (data not shown).

#### c. Ha-RAS and Rac1

In the case of HRAS^G12V^, previous studies involving Caco-H2 cells have shown that MAPK, PI3K and JUN N-terminal kinase (JNK) pathways are highly activated as compared to parental Caco-2 cells [16,25]. Similarly, in the MCF10A breast cancer cell line HRAS activates PI3K pathway through Rac1 resulting in invasive phenotype [56]. Inhibition of MAPK but not Rac1 restored E-cadherin junctions and epithelial morphology in HRAS^D12^-transfected cells [57]. Furthermore, the role of Rac1 in maintaining malignant phenotype of mouse skin tumour cells was investigated and showed that dominant negative Rac1 reduces migration, invasion and tumour growth through inhibition of MAPK signalling [58], while more recently, it was established that FAK signalling is required for TGFbeta-mediated EMT in hepatocytes [21]. In this study evidence is provided that FAK is up-regulated in Caco-H2 cells, like in invasive tumours and that Y397 phosphorylation is reduced in these cells (additional Figure 7). A previous study has shown that activated RAS induces dephosphorylation and inhibition of FAK, mediated by Fgd1-Cdc42-PAK1-MEK-ERK signaling cascade. This inhibition of FAK mediated by this signal promotes Ras-induced cell migration, invasion, and metastasis [59]. Taken together, a model for HRAS^G12V^- induced EMT is proposed in human colon cells (Figure 10D): mutant HRAS exerts its function through different pathways (MAPK, PI3K) and induces PI3K dependent Rac1 activation and expression of other EMT-mediators to contribute in EMT phenotype and related properties. Downstream of these pathways other molecules also implicated in EMT, like vimentin and integrin α6, have been shown to play a role in migration properties of these cells through a Jun/Fra1/AP-1 (activator protein-1)-dependent regulation [25,60,61].

## Conclusion

This study shows for the first time that BRAF and RAS oncogenes utilise different Rho signalling pathways to induce migration and invasion properties in human colon adenocarcinoma cells. BRAF^V600E ^provides human colon adenocarcinoma cells with a more "aggressive" phenotype and consequential migrating and invading properties, mainly through RhoA activation, regulated by MEK pathway. KRAS^G12V ^utilizes Cdc42 in order to enhance cell migration and filopodia formation, while Rac1 GTPase plays important role in HRAS^G12V^-induced EMT characteristics, both at least partially dependent on PI3K pathway. Moreover, BRAF and KRAS oncogenes cooperate with TGFβ-1 pathway to provide cells with additional transforming properties. Findings and cell models proposed here may provide useful tools for future studies that will focus on further dissection of specific oncogene induced signalling pathways. This can be later exploited toward the design of colon cancer therapeutics targeting specific Rho pathways based on the oncogenic mutations found in each patient.

## Abbreviations

EMT: epithelial to mesenchymal transition; RhoA: Ras homolog gene family member A; Rac1: Ras-related C3 botulinum toxin substrate 1; Cdc42: cell division cycle 42; ERK: extracellular signal-regulated kinase; FAK: Focal Adhesion Kinase; TGFβ-1: Transforming growth factor beta-1; GEF: guanine nucleotide exchange factor; MAPK: Mitogen-Activated Protein Kinase; PI3K: Phosphoinositide 3-kinase; JNK: JUN N-terminal kinase; MEK: mitogen-activated protein kinase/extracellular signal-regulated kinase kinase.

## Competing interests

The authors declare that they have no competing interests.

## Authors' contributions

EM carried out the cell and molecular studies, and drafted the manuscript. EO carried out gene expression analysis and participated in the design of experiments as well as in the developed of new cell lines. MK and LA participated in the development of new cell lines. TS and SS provided cell lines with silenced K-Ras oncogene. AP conceived of the study, and participated in its design and coordination and helped to draft the manuscript. All authors read and approved the final manuscript.

## Supplementary Material

Additional file 1 **Additional Figure 1**. Light microscope photographs of Caco-2, Caco-BR, Caco-K, Caco-H, HT29 and DLD-1 cells show the differences in their morphology. Click here for file

Additional file 2 **Additional Figure 2**. (A) Transient transfection of BRAF^V600E ^in Caco-2 cells by calcium phosphate followed by a GST-pull down RhoA activation assay. (**B**) Moderate effects of BRAF^V600E ^following transient transfection in Caco-2 cells with respect to the cell migration and invasion. (**C**) Arrows indicate polycarbonate membrane pore empty or occupied by cells in process of migrating or invading through. Click here for file

Additional file 3 **Additional Figure 3**. Caco-BR13 cells were treated with MEK inhibitor UO126 for 30 and 60 minutes at indicated concentrations to determine optimal conditions. Click here for file

Additional file 4 **Additional Figure 4**. Caco-H2 cell were treated with the PI3K inhibitor wortmanin for 30 and 60 minutes at indicated concentrations to determine optimal conditions. Click here for file

Additional file 5 **Additional Figure 5**. Cell proliferation of Caco-2, Caco-BR and Caco-K cells following TGFβ-1 treatment for 6 days, TGFβ-1 was refreshed every 2 days. Click here for file

Additional file 6 **Additional Figure 6**. Cross-talk of Rho GTPaes. (A) Caco-BR13 cells were treated with 80pmol siRNA for RhoA for 48 hours and GST pull-down assay was performed to examine the effect on Cdc42 activity. (**B**) Caco-K15 cells were treated with 160pmol siRNA for Cdc42 for 48 hours and expression levels of Rac1 were tested. (**C**) Caco-H2 cells were treated with 160pmol of Rac1 specific siRNA and the expression levels of RhoA were examined. Click here for file

Additional file 7 **Additional Figure 7**. Western blot analysis of FAK and pFak (Y397) (upper panel) and confocal images showing focal adhesions in Caco-2, Caco-BR Caco-K and Caco-H cells after staining with antibody against FAK. Representative images are shown. Click here for file
