# Supplementary figures and images for: BRAF and RAS oncogenes regulate Rho GTPase pathways to mediate migration and invasion properties in human colon cancer cells: a comparative study
Source: Mol Cancer. 2011 Sep 23;10:118. doi: 10.1186/1476-4598-10-118 (PMC3189908; doi:10.1186/1476-4598-10-118)

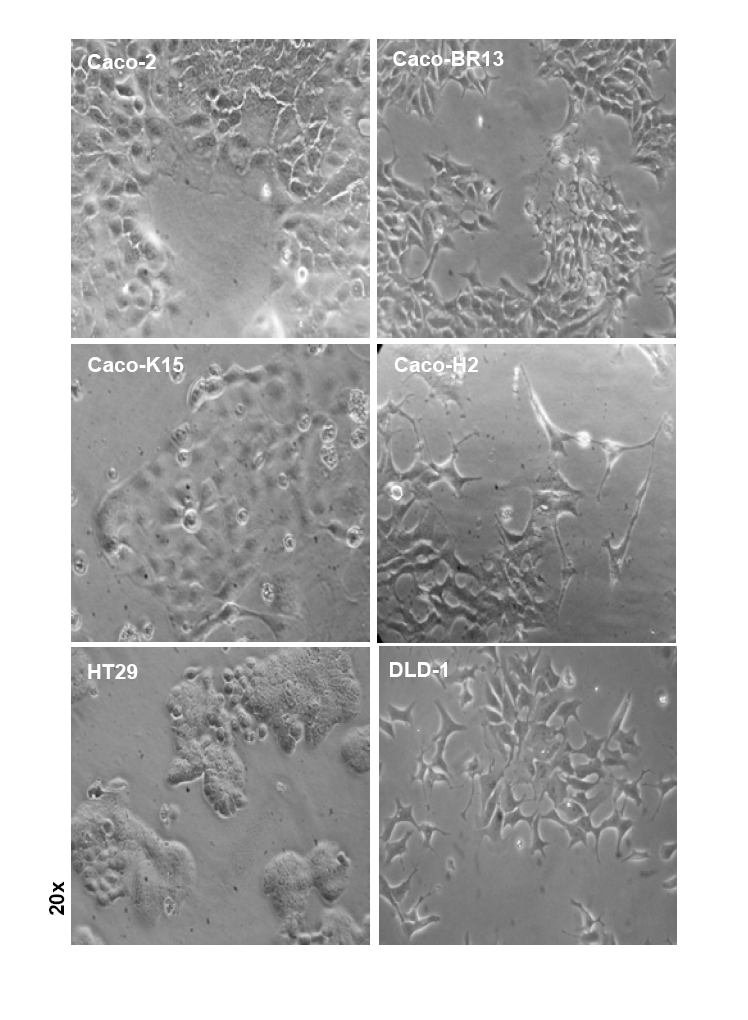

Supplement: Additional file 1 — Additional Figure 1. Light microscope photographs of Caco-2, Caco-BR, Caco-K, Caco-H, HT29 and DLD-1 cells show the differences in their morphology. [file 1476-4598-10-118-S1.JPEG]

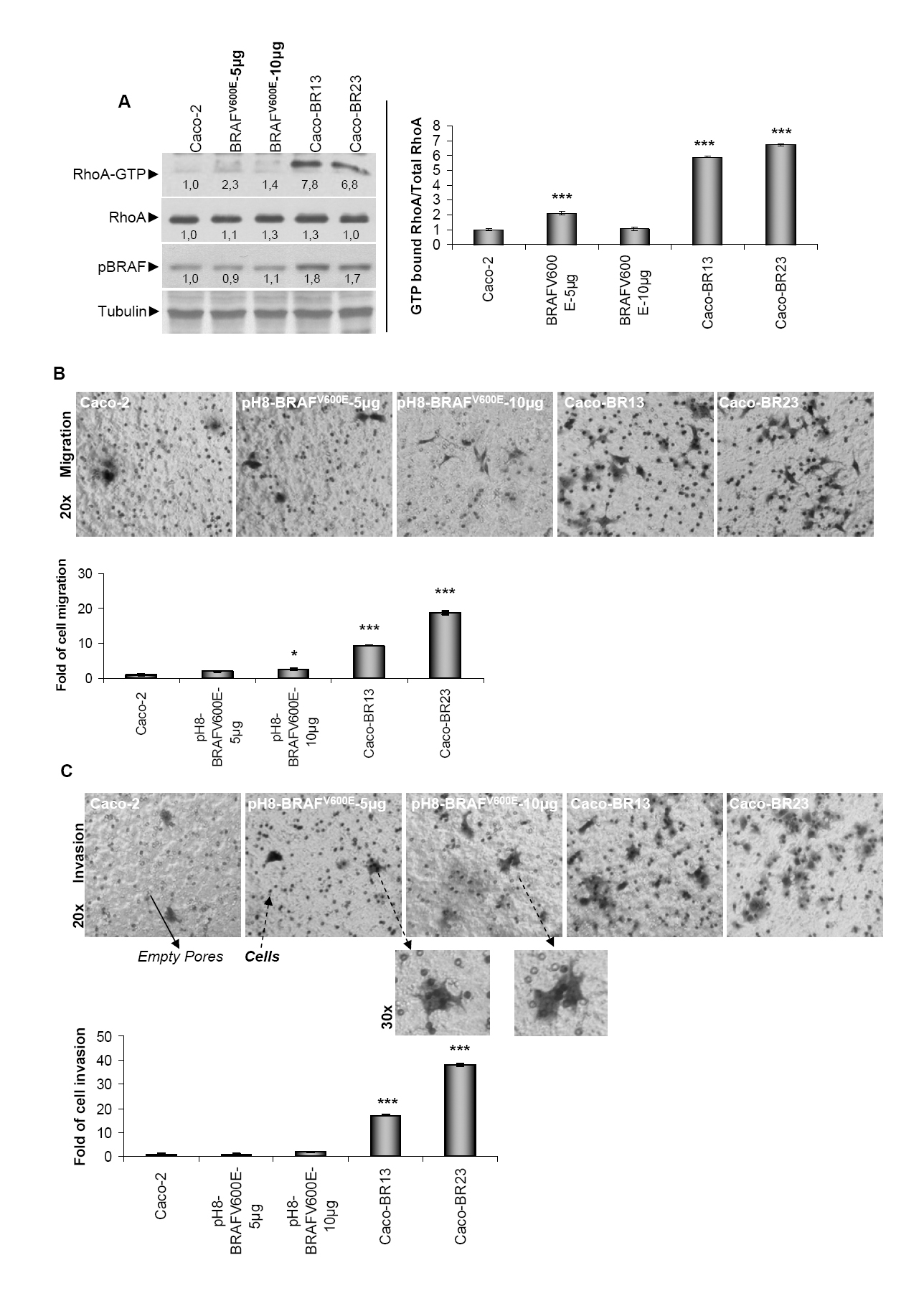

Supplement: Additional file 2 — Additional Figure 2. (A) Transient transfection of BRAFV600E in Caco-2 cells by calcium phosphate followed by a GST-pull down RhoA activation assay. (B) Moderate effects of BRAFV600E following transient transfection in Caco-2 cells with respect to the cell migration and invasion. (C) Arrows indicate polycarbonate membrane pore empty or occupied by cells in process of migrating or invading through. [file 1476-4598-10-118-S2.JPEG]

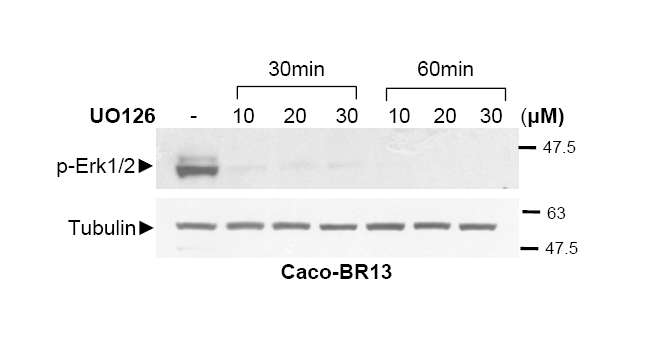

Supplement: Additional file 3 — Additional Figure 3. Caco-BR13 cells were treated with MEK inhibitor UO126 for 30 and 60 minutes at indicated concentrations to determine optimal conditions. [file 1476-4598-10-118-S3.JPEG]

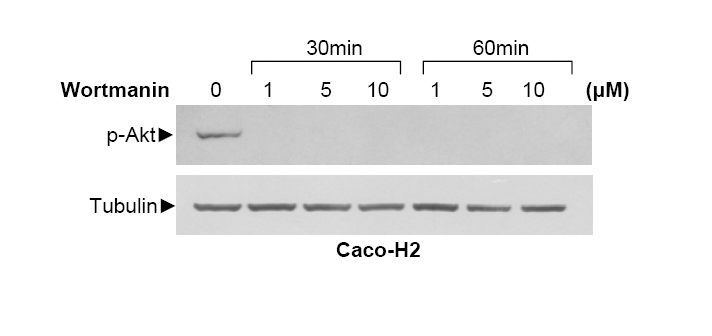

Supplement: Additional file 4 — Additional Figure 4. Caco-H2 cell were treated with the PI3K inhibitor wortmanin for 30 and 60 minutes at indicated concentrations to determine optimal conditions. [file 1476-4598-10-118-S4.JPEG]

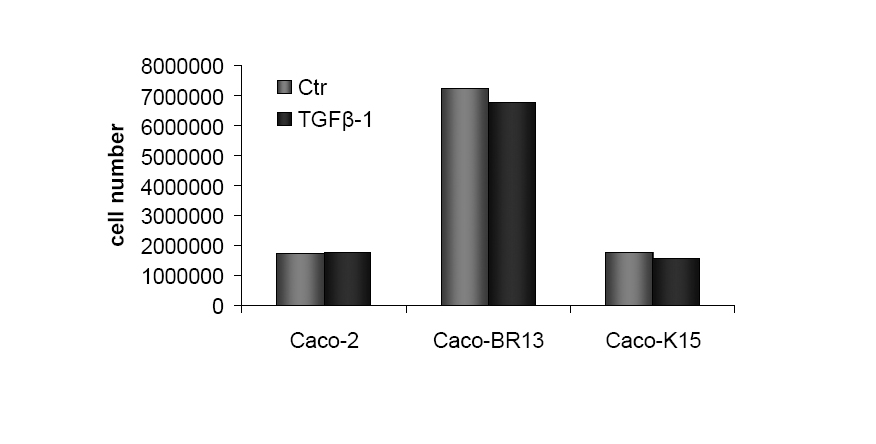

Supplement: Additional file 5 — Additional Figure 5. Cell proliferation of Caco-2, Caco-BR and Caco-K cells following TGFβ-1 treatment for 6 days, TGFβ-1 was refreshed every 2 days. [file 1476-4598-10-118-S5.JPEG]

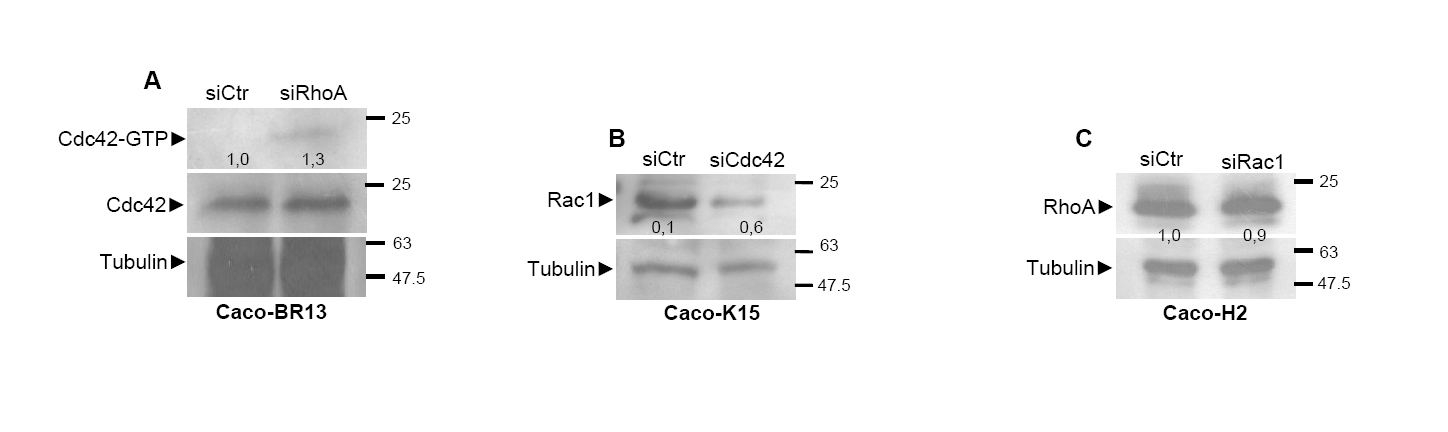

Supplement: Additional file 6 — Additional Figure 6. Cross-talk of Rho GTPaes. (A) Caco-BR13 cells were treated with 80pmol siRNA for RhoA for 48 hours and GST pull-down assay was performed to examine the effect on Cdc42 activity. (B) Caco-K15 cells were treated with 160pmol siRNA for Cdc42 for 48 hours and expression levels of Rac1 were tested. (C) Caco-H2 cells were treated with 160pmol of Rac1 specific siRNA and the expression levels of RhoA were examined. [file 1476-4598-10-118-S6.JPEG]

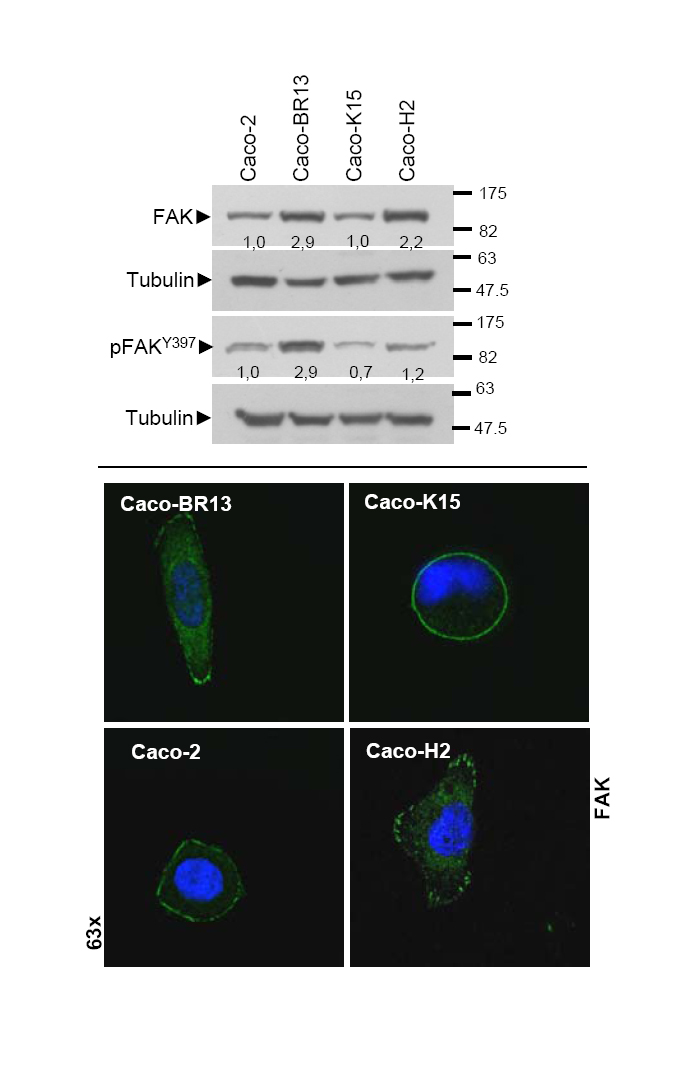

Supplement: Additional file 7 — Additional Figure 7. Western blot analysis of FAK and pFak (Y397) (upper panel) and confocal images showing focal adhesions in Caco-2, Caco-BR Caco-K and Caco-H cells after staining with antibody against FAK. Representative images are shown. [file 1476-4598-10-118-S7.JPEG]
